# Generalized peritonitis secondary to spontaneous rupture of the urinary bladder in a diabetic patient: A case report

**DOI:** 10.1016/j.ijscr.2022.107458

**Published:** 2022-07-28

**Authors:** Foolad Eghbali, Hesam Mosavari, Ahmad Madankan, Vahid Hariri, Kiana Garakani, Mansour Bhahdoust

**Affiliations:** a Minimally Invasive Surgery Research Center, Division of Minimally Invasive and Bariatric Surgery, Rasool-e Akram Hospital, Iran University of Medical Sciences, Tehran, Iran; b Rasoul Akram Hospital, Iran University of Medical Sciences, Tehran, Iran; c Student in Molecular Cell Biology and Data Science, University of California Berkeley, San Francisco, CA, USA; d Department of Epidemiology, School of Public Health, Shahid Beheshti University of Medical Sciences, Tehran, Iran

**Keywords:** Spontaneous rupture of the urinary bladder (SRUB), Laparotomy, Diabetic

## Abstract

**Introduction and importance:**

Spontaneous rupture of the urinary bladder (SRUB) secondary to emphysematous cystitis(EC) in diabetic patients is extremely rare. Clinical presentations are often non-specific and display the signs and symptoms of peritonitis. The diagnosis is usually made after exploratory laparotomy.

**Case report:**

A 70-year-old diabetic woman presented to the emergency department with sudden diffuse abdominal pain and hematuria of six hours duration. Physical examination revealed generalized peritonitis. Multi-slice abdominal and pelvic CT scans showed free air and fluid in the abdominal cavity. After proper resuscitation, the patient was transferred to the operating room for exploratory laparotomy. A 2 cm full-thickness bladder rupture was noted at the dome of the bladder, which was repaired.

**Clinical discussion:**

We noticed free air in the urinary bladder wall postoperatively in the CT scan, which is the radiological sign of EC. The Pathology result was in concordance with the diagnosis.

**Conclusions:**

SRUB in patients with poorly controlled diabetes and EC is highlighted in this case study. Urinary bladder rupture secondary to EC should be considered When a diabetic patient with a history of urinary symptoms presents with an acute onset of abdominal pain suggestive of peritonitis. Uneventful recovery from SRUB is dependent on early diagnosis and treatment.

## Highlights

• SRUB should be considered in diabetic patients with a history of urinary symptoms.
• An uneventful recovery from SRUB is dependent on early diagnosis and treatment.
• EC may be seen in diabetic patients with abdominal pain, and symptoms of cystitis.
• Proper antibiotic therapy leads to successful treatment and prevents complications.

## 1 Introduction

Spontaneous rupture of the urinary bladder (SRUB) is extremely rare. Although the cause of SRUB is unclear, in most cases, a predisposing factor makes a patient prone to bladder rupture [1]. Urinary retention, infections, vaginal delivery, vomiting, and radiation, among others, are some of the predisposing conditions associated with SRUB [2], [3], [4], [5], [6]. There are also very limited reports of SRUB in diabetic patients with EC [7]. The non-specific nature of the clinical signs and symptoms of SRUB makes it easy to misdiagnose the condition. Exploratory laparotomy is the gold standard, and most cases are diagnosed during laparotomy [1]. Open surgical bladder repair has been recommended for an intraperitoneal bladder rupture. Because of the risk of sepsis and peritonitis, prompt diagnosis and surgical intervention are warranted for SRUB [8], [9]. Herein, we present a case of SRUB in a 70-year-old diabetic female patient presenting with acute generalized peritonitis. The work has been reported in line with the SCARE 2020 criteria [10].

## 2 Case presentation

A 70-year-old woman presented to the emergency department with sudden diffuse abdominal pain and hematuria of six hours duration. The patient denied any prior history of trauma or intoxication. Her past medical history was significant for type 2 diabetes mellitus from 15 years ago, multiple episodes of urinary tract infections (UTI) in the past five years, and urinary frequency. Her drug history was only metformin, two grams per day from 15 years ago. Upon arrival, vital signs were as follows: blood pressure of 110/70 mmHg, heart rate of 100 beats per minute, respiratory rate of 18 breaths per minute, and body temperature of 38 °C.

**Fig. 1:**
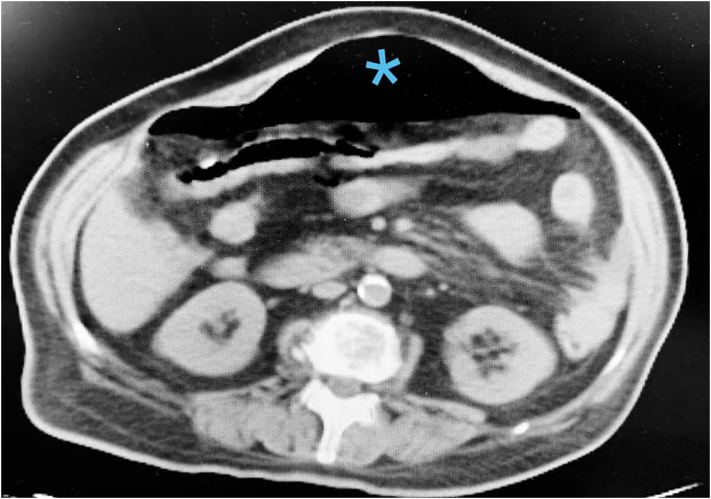
Blue mark shows the free air in the abdominal cavity.

On physical examination, generalized rebound tenderness and abdominal guarding were detected. Laboratory results were as follows: leukocytosis of 14 × 10^9^/L with neutrophilia, serum creatinine of 2 μmol/L, urea of 55 mmol/L, the elevated blood sugar of 400 mg/dL, serum potassium of 4 mmol/L, serum sodium of 130 mmol/L. Urinary catheterization drained 150 mL of bloody urine. Microscopic urinalysis revealed many RBCs, WBCs, and bacteria per high power field. Multi-slice abdominal and pelvis CT scans showed free air and fluid in the abdominal cavity and air in the bladder wall (Fig. 1). Although, the latter was noticed retrospectively after surgery. Based on these findings, the patient was diagnosed with generalized peritonitis secondary to hollow viscous perforation. After proper resuscitation with 2 l of IV crystalloids, the patient was transferred to the operating room for exploratory laparotomy by a senior general surgeon with about twenty years of experience in an academic center in a general hospital.

**Fig. 2:**
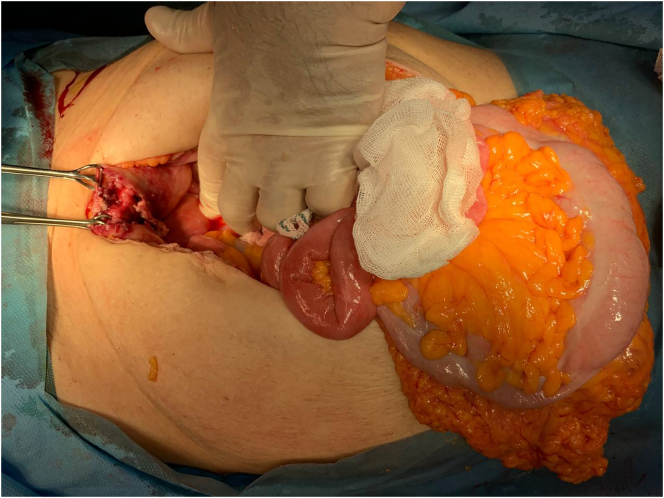
Rupture at the dome of the urinary bladder.

Under general anesthesia, the abdominal wall was opened with a midline incision. Over a liter of purulent fluid was found in the peritoneal cavity. Despite a thorough investigation, no gut perforation was found. Two large hemangiomas were found at the right and left liver lobes. A 2 cm full-thickness bladder rupture was noted at the dome of the bladder (Fig. 2). The region surrounding the rupture was thin, but the remainder of the bladder was in good condition. A bladder biopsy was collected from the perforation's edge. A two-layer closure with a 2/0 Vicryl suture was performed to repair the bladder wall rupture then a suprapubic catheter was inserted because the main catheter could get blocked due to bleeding and clot formation.

**Fig. 3:**
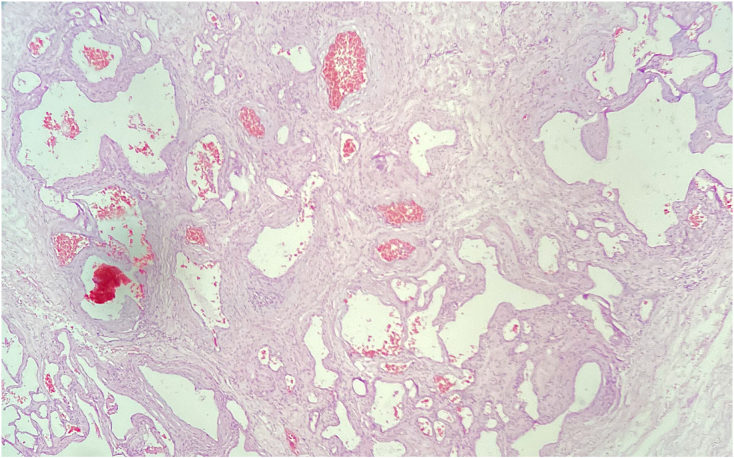
Pathology photomicrograph - the urinary bladder wall exhibited diffuse edema, hemorrhagic foci, and numerous cystic gas collections.

The postoperative course was uneventful, and she was completely cooperative. Based on postoperative re-examination of the CT scan and symptoms, the patient was diagnosed with EC. The patient completed antimicrobial therapy with intravenous ceftriaxone (based on antibiogram results that demonstrated E-coli more than 100,000 CFUs/ml) and was discharged from the hospital one week after surgery in good condition with an indwelling urinary catheter and a suprapubic catheter. Two weeks after discharge, she was returned for a follow-up visit, and the suprapubic catheter was extracted. A follow-up cystoscopy was performed one month later and noted only mild to moderate trabeculation, and there was a scar at the site of perforation. Pathology came back as transmural infiltration of mixed inflammatory cells with surface ulceration (Fig. 3). During the last follow-up (two months after surgery), she was in good condition without any abdominal and urinary signs and symptoms.

## 3 Discussion

SRUB is a rare and potentially life-threatening emergency. The incidence of SRUB is around 1 in 126,000, including less than 1 % of bladder injuries, while external trauma is the most common cause [11]. One literature review pointed out that most cases of SRUB were intraperitoneal (458 of 713 patients) and less commonly extraperitoneal (54 of 713 patients), and most of the patients were middle-aged men [1]. These findings show the difference with traumatic urinary bladder rupture, which is usually extraperitoneal [8].

The suggested cause of the SRUB is the weakening of the wall of the urinary bladder, primarily due to urine retention [1], [12]. A series of predisposing factors have been seen in patients with SRUB, including but not limited to chronic urinary retention, childbirth, lower urinary tract obstruction, alcohol abuse, bladder dysfunction, bladder surgery, diverticulum, tumors, malignancy, infection, and inflammation [1], [4], [5], [6].

Poorly controlled diabetes can lead to UTI, bladder dysfunction, and urinary retention, a major predisposing factor for SRUB [13]. Although our patient's diabetic cytopathic presented as an overactive bladder syndrome with urinary frequency in the past, we assume that our patient's voiding reflexes became sluggish over time, eventually leading to urinary retention.

Abdominal pain is the most common sign of SRUB [1]. Other common symptoms are gross hematuria and abdominal tenderness [9]. Abdominal distension, voiding problems, fever, nausea, and vomiting occur less frequently [1], [9]. These non-specific symptoms make the diagnosis of SRUB very challenging, even with the aid of CT imaging [2]. Hence, most patients are misdiagnosed with digestive system inflammatory disorders, acute abdomen, renal failure, and bladder tumor or inflammation [1].

Retrograde cystography (CT or conventional) has been recommended to evaluate bladder injuries [9], [14]. However, most reported SRUB cases were diagnosed in the operating room during a laparotomy for acute peritonitis [1]. A limited number of reports of abdominal air in CT scans have been reported, which is a classic finding of hollow viscus perforation. This finding and ascites are more common in reports of bladder rupture due to infection and inflammation. Therefore, it is recommended that the bladder be examined later if the intestines were healthy at the time of laparotomy [7], [15], [16], [17], [18], [19].

There is a lack of specific guidelines for the management of SRUB. The American Urology Association (AUA) and the European Association of Urology (EAU) recommend surgical repair for intraperitoneal bladder injuries. The AUA recommends urethral catheter drainage without suprapubic cystostomy after the surgical repair. Conservative management has not been recommended for intraperitoneal bladder injuries [8], [9], [14]. Although, a recent review of the literature found that conservative management could be successful in the absence of severe infection, bleeding, or major injuries [1]. Adequate urination drainage and antibiotics are the cornerstones of conservative treatment [20].

EC is a unique form of a complicated UTI caused by gas-forming organisms. *Escherichia coli* is the most common isolated bacteria, as in our case. Major risk factors for EC are diabetes mellitus and urinary retention. It is more commonly seen in older women in their 60s or 70s [7], [20], [21]. EC has a wide range of clinical presentations. In a review of 53 cases, the most common clinical presentation of EC was abdominal pain. At the same time, only half of the patients experienced classic symptoms of acute cystitis, including dysuria, urinary frequency, and urinary urgency [22].

**Fig. 4:**
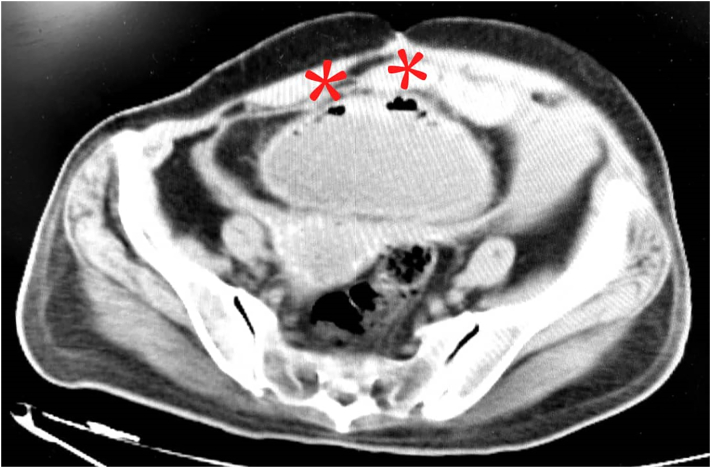
Red marks indicate gas in the bladder wall, a radiographic sign of EC. (For interpretation of the references to colour in this figure legend, the reader is referred to the web version of this article.)

The diagnosis of EC is generally made in patients found to have air within the bladder wall on abdominal imaging. Early identification of intraluminal or intramural gas in the abdomen can be achieved by abdominal CT, which is a highly sensitive modality [23]. In our case, the intramural gas could be seen on a CT scan. However, it was found postoperatively in the CT scan re-examination (Fig. 4).

Antibiotics are usually effective in treating EC. However, laparotomy is indicated in complicated EC with peritoneal signs, pneumoperitoneum, or perivesical abscess [22].

## 4 Conclusion

SRUB in patients with poorly controlled diabetes and EC is highlighted in this case study. Spontaneous rupture of the urinary bladder should be considered in diabetic patients with a history of urinary symptoms present with acute onset of abdominal pain. An uneventful recovery from SRUB is dependent on early diagnosis and treatment. EC should also be considered in diabetic patients with poor control of blood sugar, abdominal pain, and symptoms of cystitis. Proper and prompt antibiotic therapy leads to successful treatment and prevents further complications.

## Sources of funding

N/A.

## Ethical approval

The ethics committee approved this study at the Iran University of medical science.

## Consent

Written informed consent was obtained from the patient to publish this case report and accompanying images. On request, a copy of the written consent is available for review by the Editor-in-Chief of this journal.

## Research registration

N/A.

## Guarantor

Dr. Foolad Eghbali

## Provenance and peer review

Not commissioned, externally peer-reviewed.

## CRediT authorship contribution statement

- Conception and design: F E, M B, M N

- Analysis and interpretation of data: N/A

- Data collection: A V E

- Authors participate in drafting the article or revising: F E, M B, H M

## Declaration of competing interest

The authors declare that they have no competing interests.
